# Metastatic duodenal GIST: role of surgery combined with imatinib mesylate

**DOI:** 10.1186/1477-7800-4-9

**Published:** 2007-03-29

**Authors:** Kamran Mohiuddin, Saira Nizami, Asma Munir, Breda Memon, Muhammed A Memon

**Affiliations:** 1 Department of Surgery, Aga Khan University Hospital, Karachi, Pakistan; 2 Department of Surgery, Whiston Hospital, Warrington Road, Prescot, Merseyside, UK

## Abstract

**Background:**

The best possible treatment of metastatic high grade large duodenal GIST is controversial. Surgery (with or without segmental organ resection) remains the principal treatment for primary and recurrent GIST. However, patients with advanced duodenal GIST have a high risk of early tumour recurrence and short life expectancy.

**Method:**

We present a case of a young man treated with a combined modality of surgery and imatinib for an advanced duodenal GIST.

**Results:**

He remains asymptomatic and disease free 42 months following this combined approach.

**Conclusion:**

Treatment with imatinib has dramatically improved the outlook for patients with advanced, unresectable and/or metastatic disease.

## Background

Gastrointestinal stromal tumours or GISTs are the most common mesenchymal neoplasms of the gastrointestinal tract demonstrating positive c-kit (CD117) immunohistochemical staining. Approximately 50–70% originates in the stomach whereas 20–30% of from the small bowel, with duodenum being the least common site. Less frequent sites include the colon and rectum (5–15%) and esophagus (< 5%). These tumours usually grow submucosally but may also manifest as exophytic extraluminal subserosal growth. We report a case of a young man with a large extraluminal advanced duodenal GIST treated successfully with combination of surgery and imatinib mesylate.

## Case report

**Figure 1:**
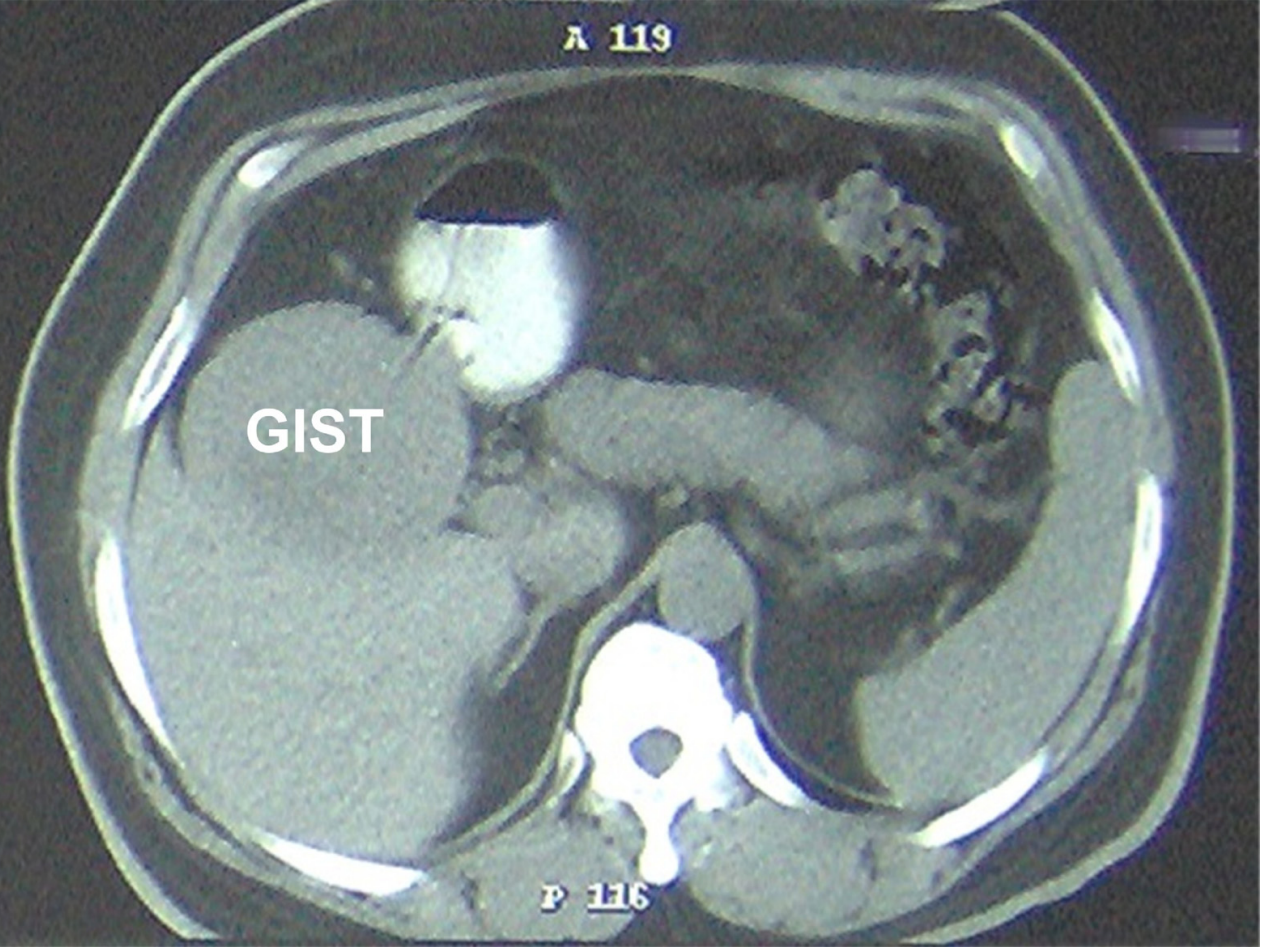
CT scan of patient showing duodenal GIST.

**Figure 2:**
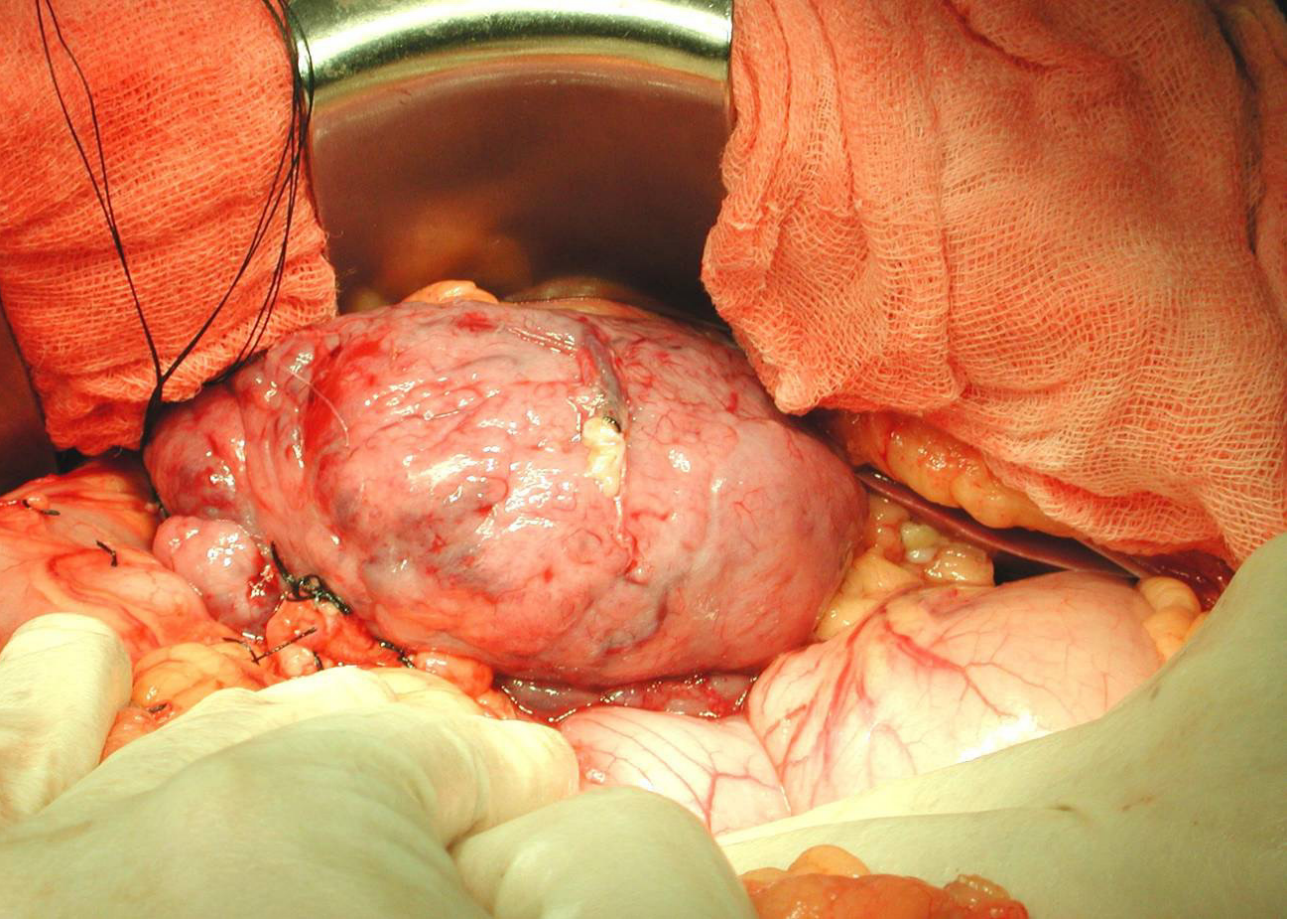
Intra-operative view of the duodenal GIST.

**Figure 3:**
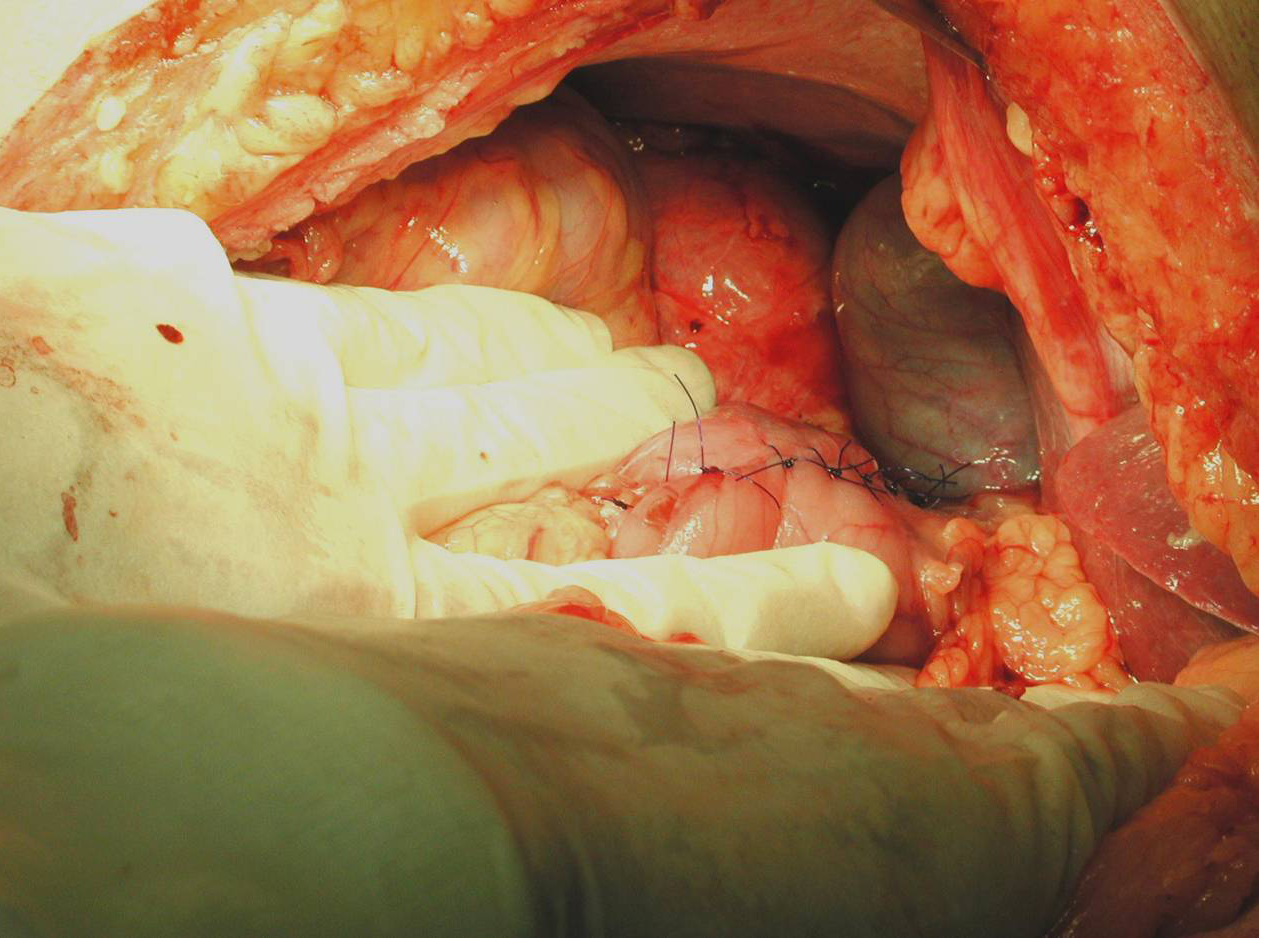
Post resectional view with primary closure of the duodenum.

**Figure 4:**
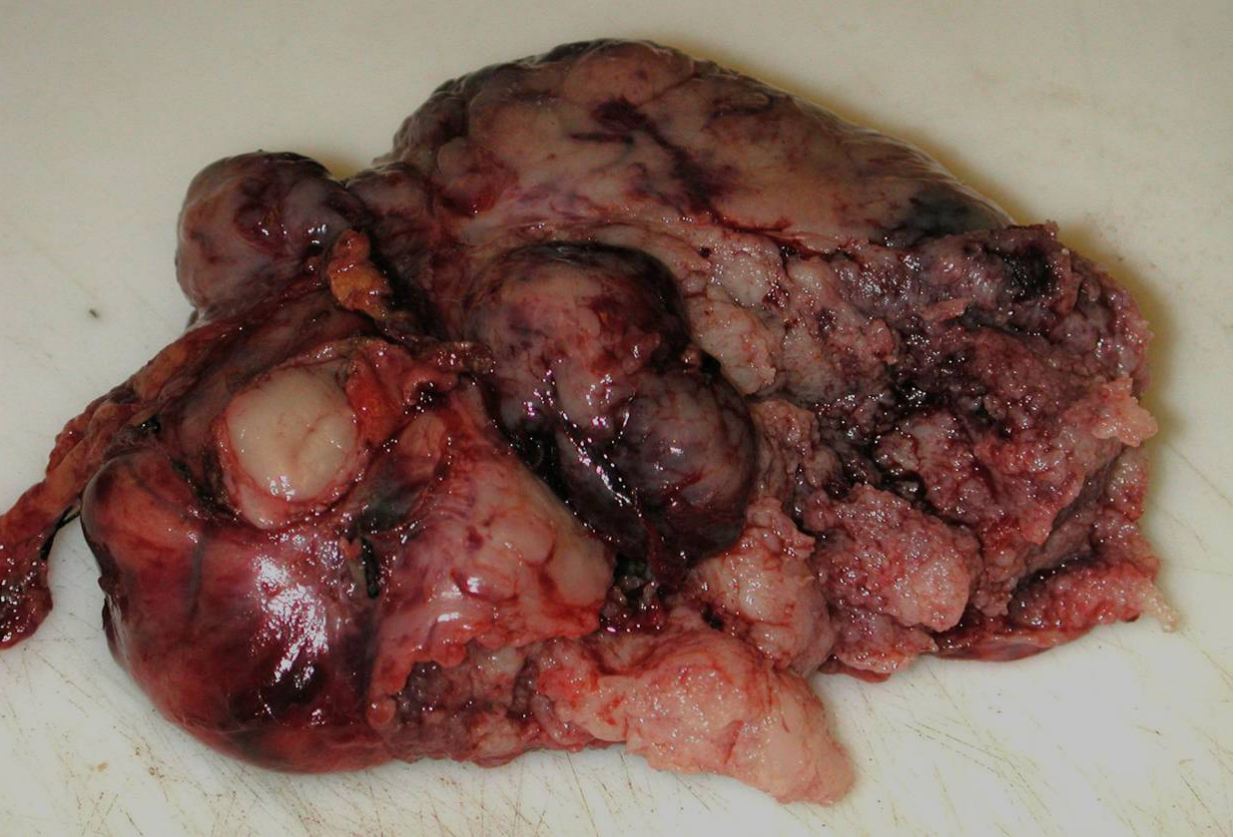
Macroscopic appearance of duodenal GIST specimen.

A 56 year old man, who was on regular aspirin following coronary artery bypass surgery, presented with an eight month history of intermittent malaena requiring an emergency admission to the hospital. At the time of admission the only positive finding was that of pallor. His full blood count revealed microcytic anaemia with haemoglobin of 7 g/dl. He was therefore transfused 4 units of packed red cells. He underwent an urgent upper gastrointestinal endoscopy which revealed a bulge in the 2^nd ^part of the duodenum without any visible mucosal abnormality or intraluminal blood. The duodenal biopsies revealed mild duodenitis and the CLO test for Helicobacter Pylori was negative. A computerized tomography scan of the abdomen demonstrated a well demarcated enhancing 9.5 × 9.0 cm mass arising from the lateral wall of the 2^nd ^part of the duodenum without any intra-abdominal lymphadenopathy or liver metastases (Figure 1). A provisional diagnosis of duodenal GIST was entertained. The patient underwent an elective exploratory laparotomy which revealed a 10 × 10 cm fleshy friable multi-lobulated exophytic mass arising from the anterior wall of the 2^nd ^part of duodenum on a narrow pedicle (Figure 2). Tumour deposits were also seen on the adjacent mesocolon. A wedge excision of the antimesenteric portion of the duodenum containing the pedicle was performed (Figure 3, 4). A frozen section of the duodenum was obtained to confirm tumour free margins before primarily closing it with 2/0 Vicryl in an interrupted fashion. Also the adjacent mesocolon containing tumour deposits was also excised and the defect closed with interrupted sutures. The patient's postoperative recovery was uneventful and he was discharged home seven days later. Histopathology revealed that the tumour consists of spindle cells which in areas were arranged in fascicles. There was associated haemorrhage and the cells exhibited moderate pleomorphism. Furthermore the duodenal margins were free from tumour (R0 resection). Immunohistochemistry was strongly positive for CD117. Moreover, the mesocolon deposits consists of spindle shaped cells once again positive for CD117. The final diagnosis was that of a high grade metastatic gastrointestinal stromal tumour (GIST).

Because of size of the tumour, histopathological and immunochemistry findings and the presence of tumour deposits in the adjacent mesocolon, the patient was given two years of imatinib mesylate therapy. A follow-up CT scans of chest, abdomen and pelvis have failed to reveal any evidence of recurrence. The patient is still alive and well without any signs of recurrence 42 months following this treatment.

## Discussion

Duodenal GIST can present with vague and non-specific symptoms such as upper abdominal pain (50% to 70%), gastrointestinal haemorrhage (20% to 50%) and an abdominal mass [1,2]. A patient presenting with gastrointestinal haemorrhage may reveal a submucosal mass on endoscopy and biopsies are diagnostic in only 50% of cases [3]. In our patient the endoscopy and biopsies were not helpful because the tumour was subserosal. The CT scan however raised the suspicion of duodenal GIST.

At laparotomy a large duodenal tumour on a narrow pedicle was encountered. It was felt that a wedge duodenal resection would be adequate provided tumour free margins could be obtained. A frozen section confirmed histologic tumour-free margins. Furthermore the adjacent mesocolon containing tumour deposit was also excised.

There is a wide variation in the clinical behaviour of gastrointestinal GIST's, most being benign. However, small bowel (jejunum, duodenum and the ileum) GISTs which constitute 20% to 30% of gastrointestinal stromal tumours have a high propensity for malignant behaviour [4]. The factors used in evaluation of GIST are summarized in Table 1[5-10]. Certainly larger size tumours with high mitotic figures, cytological atypia, necrosis and pleomorphism favours malignancy. Our patients had all such features along with implantation over the adjacent mesocolon and therefore it was considered not only high grade but metastatic in nature.

**Table 1:** Parameters used in evaluation of GIST malignancy

**Authors (year)**	**Site**	**Size**	**Mitosis**	**Histological Type**	**Cytological Atypia**	**Necrosis**
Amin MB et al (1993)^5^	-	+	+	-	-	-
Kindblom et al (1998)^6^	-	-	+	-	+	+
Fletcher et al (1998)^7^	-	-	+	+	+	-
Panizo et al (2000)^8^	-	+	+	-	-	-
Trupiano et al (2002)^9^	-	+	+	+	+	-
Miettinen et al (2002)^10^	+	+	+	-	-	-

The overall 5 year survival for completely resected GISTs ranges from 30 – 80% [11]. The relapse rate for patients having surgery ranges from 5% in those that have a complete resection, to 90% in those with unresectable and/or metastatic disease. The median survival for patients with unresectable and/or metastatic disease is 12 months (ranging from 2–20 months) [12]. Patients with advanced stage disease have shown that the disease progresses after approximately 1.5 months without effective therapy [13].

Imatinib mesylate, a specific tyrosine kinase inhibitor, is one of the first targeted molecular therapies, and works by disrupting specific aspects of tumour growth. This treatment has revolutionised the treatment of unresectable and/or metastatic GIST. Following the successful treatment of a Finish patient with imatinib in a chemotherapy-resistant metastatic GIST [14], a number of studies have shown encouraging results with imatinib. In a large randomized multicentre trial conducted by Demetri et al [15], imatinib induced a sustained objective response in over half of patients with advanced unresectabe or metastatic GIST. The estimated one year survival was 88%. A number of other trials [16-19] similarly have shown good response rate and survival in patients with advanced GIST (Table 2).

**Table 2:** Summary of Imatinib Trials

**Trial**	**Source**	**Phase**	**Patients no**	**Imatinib Dosage**	**Follow-up**	**Response Rate**
Multicentre Randomized USA Trial	Demetri et al NEJM 2002^15^	II	147	400 mg od 600 mg od	34 months median	CR 1% PR – 67% SD – 16% Survival – median NR
EORTC, ISG and AGITG	Van Glabbeke M et al Eur J Cancer^16,17^	III	946	400 mg od 400 mg bid	17 months median	CR – 6% PR – 45% SD – 33% Survival – median NR
The Sarcoma Intergroup	Rankin et al Journal of Clinical Oncology 2004^18^	III	746	400 mg od 800 mg od	24 months median	CR – 3% PR – 45% SD – 26% Survival – median NR
EORTC	Van Oosterum, Lancet 2001^19^	I/II	40	Dose finding 400–1000 mg od	9–13 months	PR – 53% SD – 16%

CR – complete response, NR – Not reached, PR – partial response, SD – stable disease

We felt that our patient being at an increased risk of recurrence because of his operative and histological findings, would need adjuvant treatment with imatinib (a) to stabilise his disease and (b) to prolong his recurrence-free period and survival. He was therefore started on 400 mg of imatinib once daily. He was followed up at six monthly intervals with a full body CT scan for the first two years. Surprisingly enough, he did not suffer from any major side effects from imatinib at all. At two years imatinib was stopped and further CT scans were performed at six monthly intervals which have not shown any evidence of recurrence and the patient remains asymptomatic and disease free at 42 months following his treatment. The future plan is to monitor him closely with CT scans for another eighteen months.

## Conclusion

We feel that imatinib, a targeted tyrosine kinase inhibitor treatment in combination with surgery, has prolonged our patient's disease free survival and therefore have provided an effective adjuvant treatment for the metastatic duodenal GIST.

## Conflict of interest/funding

The author(s) declare that they have no competing interests.
